# Enhanced in vivo and ex vivo thrombin generation after lower-leg trauma, but not after knee arthroscopy

**DOI:** 10.1186/s12959-023-00493-4

**Published:** 2023-04-28

**Authors:** Carolina E. Touw, Banne Nemeth, Raymond A. van Adrichem, Astrid van Hylckama Vlieg, Rob G. H. H. Nelissen, Ton Lisman, Suzanne C. Cannegieter

**Affiliations:** 1 Department of Clinical Epidemiology, Leiden University Medical Center, PO Box 9600, 2300 RC Leiden, The Netherlands; 2 Department of Orthopaedics, Leiden University Medical Center, Leiden, The Netherlands; 3 Department of Surgery, Surgical Research Laboratory, University Medical Center Groningen, University of Groningen, Groningen, The Netherlands; 4 Department of Internal Medicine, Division of Thrombosis and Haemostasis, Leiden University Medical Center, Leiden, the Netherlands

**Keywords:** Leg injury, Knee injury, Arthroscopy, Coagulation, Thrombin generation

## Abstract

**Background:**

There is room for improvement of prevention of venous thromboembolism (VTE) after lower-leg cast application or knee arthroscopy. Information about the mechanism of clot formation in these patients may be useful to identify new prophylaxis targets. We aimed to study the effect of 1) lower-leg injury and 2) knee arthroscopy on thrombin generation.

**Methods:**

A cross-sectional study was conducted using plasma samples of POT-(K)CAST trials to measure ex vivo thrombin generation (Calibrated Automated Thrombography [CAT]) and plasma levels of prothrombin fragment 1 + 2 (F1 + 2), thrombin-antithrombin (TAT), fibrinopeptide A (FPA). Plasma was obtained shortly after lower-leg trauma or before and after (< 4 h) knee arthroscopy. Participants were randomly selected from those who did not develop VTE. For aim 1, samples of 88 patients with lower-leg injury were compared with 89 control samples (i.e., preoperative samples of arthroscopy patients). Linear regression was used to obtain mean differences (or ratios if ln-retransformed because of skewedness) adjusted for age, sex, body mass index, comorbidities. For aim 2, pre- and postoperative samples of 85 arthroscopy patients were compared, for which mean changes were obtained.

**Results:**

In patients with lower-leg injury (aim 1), endogenous thrombin potential, thrombin peak, velocity index, FPA and TAT were increased as compared with controls. In arthroscopy patients (aim 2), pre- and postoperative levels were similar for all parameters.

**Conclusion:**

Lower-leg trauma increases thrombin generation both ex vivo and in vivo, in contrast to knee arthroscopy. This may imply that the pathogenesis of VTE is different in both situations.

**Supplementary Information:**

The online version contains supplementary material available at 10.1186/s12959-023-00493-4.

## Introduction

Deep vein thrombosis and pulmonary embolism are two manifestations of venous thromboembolism (VTE), comprising pathological thrombus formation associated with long-term morbidity and mortality [1–3]. Two conditions in which patients are at increased risk of VTE, that are associated with tissue damage to the lower-leg, are trauma and knee arthroscopic surgery. According to a recent meta-analysis, the 3-month VTE risk following lower-leg cast application and knee arthroscopy is 2% and 0.6%, respectively [4]. It has become clear that the currently applied thromboprophylaxis strategy, i.e. administration of low-molecular-weight-heparin (LMWH), is not optimal [5, 6]. Hence, thromboprophylaxis needs to be improved. Information about the pathophysiological mechanism of thrombus formation in both situations could provide new prevention targets and provide a basis for better prophylaxis strategies [4].

Elevated thrombin generation potential appears to be associated with VTE risk related to major trauma [7, 8]. Whether patients with minor trauma such as of the lower-leg also have alterations in this capacity is unknown. Thrombin generation potential is quantified using in vitro (or ex vivo) thrombin generation assays (TGA), which have been extensively studied in various patient populations [9, 10]. The actual thrombin generation in vivo can be estimated by measuring plasma levels of prothrombin fragment 1 + 2 (F1 + 2): a peptide split off at prothrombin-thrombin conversion [11, 12]. Thrombin-antithrombin complexes (TAT complexes; formed at inactivation of thrombin by binding to antithrombin) is another markers of in vivo thrombin generation. Fibrinopeptide A (FPA; split off at fibrinogen-fibrin conversion) has also been used as a marker for thrombin and fibrin generation [13].

The objectives of our study were to measure the effect of (1) lower-leg injury and (2) knee arthroscopy on ex vivo thrombin generation (potential) and in vivo thrombin generation.

## Methods

### Study population

Participants of the POT-CAST (Prevention of Thrombosis following CAST immobilization) and the POT-KAST (Prevention of Thrombosis following Knee Arthroscopy) trials were included. These patients had been treated with lower-leg cast immobilization (POT-CAST) or undergone knee arthroscopy (POT-KAST). Details of these randomized controlled trials have been published previously [5]. In short, the effectiveness of low-molecular-weight heparin (LMWH) as thromboprophylaxis, compared to no therapy, was studied in both study populations by evaluating the 90-day incidences of symptomatic VTE. In both trials, participants were included between March 2012 and January 2016. Patients with a traumatic injury below the knee which required lower-leg cast immobilization (for at least 1 week) and patients scheduled for elective knee arthroscopic surgery were eligible for inclusion. Only individuals who were aged 18 years or older and did not meet any of the exclusion criteria were included. Exclusion criteria were: history of VTE, current use of anticoagulant therapy (except antiplatelet medication), contra-indications for use of LMWH, pregnancy, mental or physical disability to fulfil study requirements or insufficient knowledge of the Dutch language. Participants in both trials were asked to complete a questionnaire on putative thrombotic risk factors for VTE. In addition, blood was drawn. In POT-CAST participants, blood samples were collected upon presentation at the Emergency Department. In the majority of the participants, this took place shortly after lower-leg trauma, i.e., on the same day as the trauma occurred. In POT-KAST participants, two blood samples were provided: one sample before surgery (T0, i.e., within four hours preoperatively) and one sample after surgery (T1, i.e., within four hours postoperatively). In both patient groups, all samples were collected before thromboprophylaxis (LMWH) was administered, which also applied to the postoperative samples in the POT-KAST participants. Occurrence of symptomatic VTE was evaluated using questionnaires sent to the participants during follow-up in the trial. Additionally, participants were contacted by telephone after three months to ask whether they had been examined for a suspected VTE. Suspected symptomatic VTE was confirmed using compression ultrasound (DVT) or spiral CT scan (PE). Written informed consent was received from all participants. Both trials were approved by the Medical Ethics Committee of Leiden University Medical Center.

### Current study

**Fig. 1:**
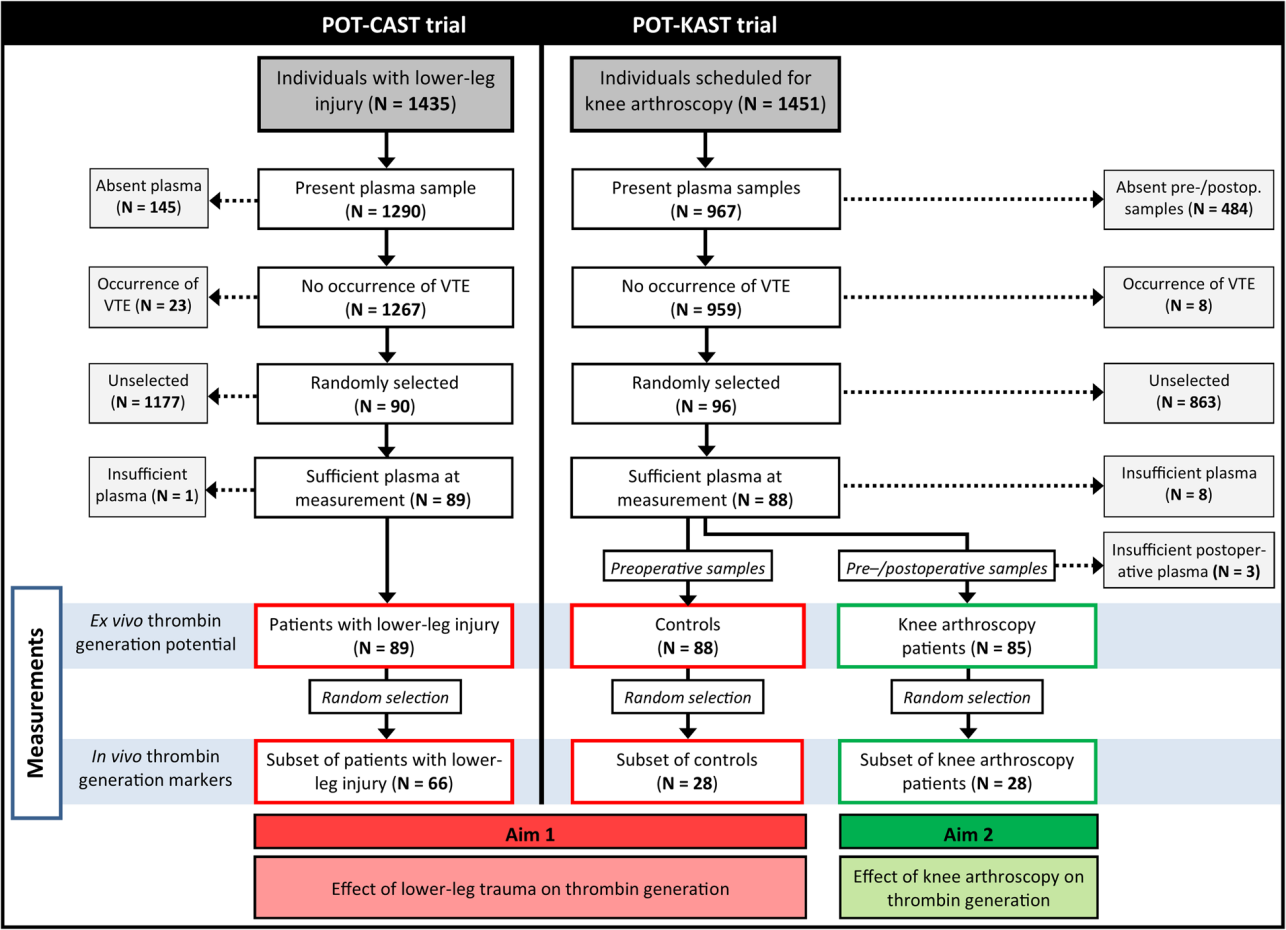
Flowchart patient selection from POT-CAST and POT-KAST trials

In total, 1435 and 1451 individuals were included in the POT-CAST and POT-KAST trials, respectively. For the current study, only those participants of the two POT-(K)CAST study populations who did not develop VTE within the first three months and had blood sample(s) available were eligible for random selection. The reason for this was to avoid distortion of the association between lower-leg injury or knee arthroscopy and thrombin generation by a certain predisposition to VTE. Details of these selections are included in the flowchart (Fig. 1).

For the first aim, i.e., to explore the effect of lower-leg trauma on ex vivo and in vivo thrombin generation, plasma samples of 89 randomly selected individuals with lower-leg injury (POT-CAST) were used, further referred to as “patients with lower-leg injury”. As control samples we used the preoperative plasma samples (i.e. at baseline or normal state) of 88 randomly selected knee arthroscopy patients (POT-KAST), further referred to as “control subjects”. We deemed these samples suitable as control samples, since they were processed and stored in the same way and thrombin generation measurements were performed at the same time. Moreover, these samples were collected before knee arthroscopy and not in the acute phase after a trauma. The predominant indication for knee arthroscopy was a meniscectomy. This surgery is performed in patients with persisting complaints after minor knee trauma in the past for which physical therapy proved inadequate. This means that at the time of blood sampling, no acute trauma was present which could have affected thrombin generation estimates.

For the second aim, i.e., to explore the effect of knee arthroscopy on ex vivo and in vivo thrombin generation, pre- and postoperative plasma samples of 85 patients out of the 88 randomly selected knee arthroscopy patients were compared. These 85 patients are further referred to as “knee arthroscopy patients”. The remaining three patients had to be excluded for these analyses due to insufficient quality of the postoperative plasma sample.

### Outcomes

Thrombin was measured both ex vivo (or in vitro) and in vivo. Ex vivo thrombin generation potential was measured by the Calibrated Automated Thrombography assay and expressed by the following parameters: lag time, peak height, endogenous thrombin potential (ETP, indicates the area under the curve) and velocity index. To obtain an indication of the extent of in vivo thrombin and fibrin generation, plasma levels of F1 + 2, TAT and FPA were measured.

### Blood collection and laboratory measurements

Blood was collected through vena puncture in the antecubital vein and drawn into 0.105 M (3.2%) vacuum citrate tubes. From these tubes, plasma was aliquoted after centrifugation during 10 min at 2500G at 18 °C. Within 4 h after vena puncture, plasma was stored at -80 °C. Ex vivo thrombin generation was measured using the CAT assay (Calibrated Automated Thrombography®; Diagnostica Stago, Asnieres, France), which is a fluorimetric method [14]. Prior to analyses, plasma was centrifuged again at 10.000 × g for 10 min as described [15]. Measurements were performed based on protocols of Thrombinoscope BV (Maastricht, the Netherlands). Coagulation was initiated by 5 pM tissue factor (Innovin, Siemens Healthineers, The Hague, The Netherlands) in the presence of 4 mM phospholipid vesicles (PS/PC/PE, 20/40/40, Avanti Polar Lipids, Alabaster, Al, USA), and soluble thrombomodulin (10 mM, Synapse B.V., Maastricht, The Netherlands). A thrombin calibrator and fluorogenic substrate from Diagnostica Stago were used. F1 + 2, TAT, and FPA were measured using commercially available enzyme-linked immunosorbent assays: F1 + 2 and TAT from Siemens Healthcare Diagnostics (The Hague, The Netherlands) and FPA from Bio-Techne (Abingdon, United Kingdom). All measurements were performed according to the manufacturer’s instructions.

### Statistical analysis

#### Aim 1: effect of lower-leg injury on thrombin generation

Ex vivo and in vivo thrombin generation were compared between patients with lower-leg injury and controls. Outcomes were expressed by means with standard deviations per patient group. Linear regression was used to obtain mean differences with 95% confidence intervals (95%CIs), which were adjusted for the following confounders: sex, age, body mass index (BMI) and comorbidities. Comorbidities included Chronic obstructive pulmonary disease (COPD), liver disease, kidney disease, rheumatoid arthritis, multiple sclerosis, heart failure, haemorrhagic stroke and arterial thrombosis. Mean differences were additionally adjusted for time of blood sampling (on a continuous scale in minutes counted from midnight), in order to correct for diurnal variation. If data were not normally distributed, they were transformed using natural logarithms (*ln*), resulting in geometric means (with 95%CIs) and mean ratios (instead of mean differences). Exact details of these calculations are included in the Supplement. Additionally, ETP was stratified for injury types grouped into: soft tissue injuries (including Achilles’ tendon rupture, ankle distortion and contusion), foot fractures (i.e. phalanx, metatarsal, tarsal) and ankle or tibia (and fibula) fractures [16]. To screen what the effect was of time between trauma and blood sampling, we plotted this against levels of ETP and TAT in scatterplots. Furthermore, we composed a thrombin generation curve for each patient group by plotting the mean amount of generated thrombin in time. This means that at each timepoint, the mean of the amount of thrombin generated was calculated, as described elsewhere [17]. Finally, in our previous analysis we found lower-leg injury to be associated with increased plasma levels of factor (F)VIII, IX, XI and fibrinogen [18]. Therefore, we included these factors in the linear regression models as independent variables to establish to what extent they explained an effect on ETP, thrombin peak and velocity index (i.e., mediation analyses).

#### Aim 2: effect of knee arthroscopy on thrombin generation

Pre- and postoperative thrombin generation estimates were expressed as means with standard deviations, or geometric means with 95%CIs if ln-retransformed due to skewedness. Paired mean changes between pre- and postoperative outcomes were calculated along with their 95%CIs. In order to adjust mean changes for diurnal variation, linear mixed models (LMMs) were employed in which time of blood sampling both pre- and postoperatively (i.e., at T0 and T1) was included on a continuous scale. We visualized the amount of generated thrombin against time in the same way as for the first aim.

All analyses were performed using Stata 16.0 (http://www.stata.com). Figures were composed using GraphPad Prism version 9.0.1 (GraphPad Software, San Diego, California USA, http://www.graphpad.com).

### Sample size considerations

For sample size calculations, we aimed to achieve a power of 80% and a level of significance of 0.05 (two-sided). Calculations were based on numbers derived from studies including orthopaedic patients and patients operated for lower extremity fractures, since more comparable studies were not available [19, 20]. We anticipated an observed mean difference/change of approximately 31.0 nM with a standard deviation (SD) of 69.0 nM for thrombin peak (height), which resulted in a sample size of at least 156 patients in total for aim 1 and 41 patients for paired measurements for aim 2. For TAT, we anticipated an observed mean difference/change of approximately 3.0 ug/l with a SD of 5.0 ug/l. This resulted in a sample size of at least 88 patients in total for aim 1 and 25 patients for paired measurements for aim 2. In vivo markers of thrombin generation such as TAT were quantified in smaller subsets, which were randomly selected as depicted in Fig. 1.

## Results

### Aim 1: Effect of lower-leg injury on thrombin generation

#### Study population

As shown in Table 1, patients with lower-leg injury were comparable to control subjects in terms of sex (57% versus 51% were male) and age (median of 55 years versus 51 years) distributions. However, comorbidities were clearly different between the groups: patients with lower-leg injury almost twice as often had comorbidities, i.e., 18% compared with 9% in controls. Most lower-leg injuries involved foot (60%) and ankle fractures (33%). In the majority of patients, blood was sampled on the same day as the trauma occurred (70%). General characteristics were similar in the smaller subsets of both groups in which in vivo thrombin generation was measured, as also shown in Table 1.

**Table 1:** General characteristics of study populations

	**Patients with lower-leg injury, available for:**		**Control subjects, available for:**	
	Ex vivo thrombin generation **(*N* = 89)**	In vivo thrombin generation **(*N* = 66)**	Ex vivo thrombin generation **(*N* = 88)**	In vivo thrombin generation **(*N* = 28)**
**Sex**				
Male, *n (%)*	51 (57.3)	38 (57.6)	45 (51.1)	15 (53.6)
**Age**				
*Median in years (IQR)*	54.7 (43.4 – 60.8)	54.8 (46.0 – 60.7)	51.0 (42.0 – 59.0)	51.0 (42.3 – 58.5)
**Body Mass Index (BMI)**				
< 20 kg m^−2^, *n (%)*	1 (1.1)	0 (0.0)	3 (3.4)	1 (3.6)
20—25 kg m^−2^, *n (%)*	28 (31.8)	19 (29.2)	25 (28.7)	8 (28.6)
25—30 kg m^−2^, *n (%)*	39 (44.3)	30 (46.2)	36 (41.4)	11 (39.3)
> 30 kg m^−2^, *n (%)*	20 (22.7)	16 (24.6)	23 (26.4)	8 (28.6)
**At least one comorbidity**				
Yes, *n (%)*	16 (18.2)	12 (18.5)	8 (9.2)	1 (3.6)
**Infection last 2 months**				
Yes,* n (%)*	8 (9.4)	7 (11.1)	11 (12.6)	6 (21.4)
**Smoking**				
Yes: currently, *n (%)*	20 (23.0)	18 (28.1)	18 (20.7)	7 (25.0)
Yes: formerly, *n (%)*	30 (34.5)	25 (39.1)	35 (40.2)	11 (39.3)
**Current use oral contraceptives**^a^				
Yes, *n (%) of women*	3 (8.3)	1 (3.7)	4 (9.8)	3 (23.1)
**Malignancy last year**				
Yes, *n (%)*	1 (1.1)	1 (1.5)	1 (1.1)	0 (0.0)
**ABO-blood type**				
Homozygote non-O, *n (%)*	6 (7.2)	5 (8.2)	12 (14.0)	1 (3.7)
Heterozygote O, *n (%)*	42 (50.6)	31 (50.8)	41 (47.7)	14 (51.9)
Homozygote O, *n (%)*	35 (42.2)	25 (41.0)	33 (38.4)	12 (44.4)
**Factor V Leiden**				
Yes: heterozygote, *n (%)*	3 (3.6)	2 (3.3)	6 (7.0)	2 (7.4)
No, *n (%)*	80 (96.4)	59 (96.7)	80 (93.0)	25 (92.6)
**Type of lower-leg injury**				
Ankle distortion, *n (%)*	1 (1.1)	1 (1.5)	NA	NA
Contusion, *n (%)*	1 (1.1)	0 (0.0)		
Achilles’ tendon rupture, *n (%)*	5 (5.7)	2 (3.0)		
Foot fracture, *n (%)*	53 (59.6)	42 (63.6)		
Ankle fracture, *n (%)*^b^	29 (32.6)	21 (31.8)		
**Surgical treatment of injury**				
Yes, *n (%)*	12 (13.5)	9 (13.6)	NA	NA
**Time between trauma and blood draw**				
Within 24 h, *n (%)*	62 (70.5)	45 (69.2)	NA	NA
Within 7 days, *n (%)*	21 (23.9)	17 (26.2)		
After 7 days, *n (%)*	5 (5.7)	3 (4.6)		
**Administration of prophylactic LMWH (after blood draw)**				
Yes, *n (%)*	42 (47.2)	31 (47.0)	48 (54.5)	14 (50.0)

*IQR* Interquartile range (25^th^-75^th^ percentile)

^a^Including hormonal therapy

^b^Ankle fractures: infrasyndesmotic (*n* = 11), transsyndesmotic (*n* = 11), suprasyndesmotic (*n* = 3) and unclassified (*n* = 4)

#### Outcomes

All thrombin generation parameters and plasma levels in patients with lower-leg injury are shown in Table 2, with the following means: ETP 566.0 nM IIa *min, thrombin peak 152.3 nM IIa, lag time 2.0 min, velocity index 72.8 nM IIa/min. As shown in Fig. S1, stratification for injury type showed that the median ETP was clearly higher in patients with fractures than with soft tissue injuries. However, there was only a minimal difference between small (i.e., foot) and large (i.e., ankle/tibia and fibula) fractures. Regarding the in vivo thrombin generation markers, (geometric) mean levels were TAT 5.7 ug/L, F1 + 2 260.7 pmol/L and FPA 143.9 ng/mL. A longer interval between lower-leg trauma and blood draw did not affect ETP nor TAT, as shown in Fig. S2.

**Table 2:** Aim 1: ex vivo and in vivo thrombin generation compared between patients with lower-leg injury and controls

	**Mean (SD)**^a^		**Mean difference/ratio (95%CI)**^a^	**Adj. mean difference/ratio (95%CI)**^a,b^	**Adj. mean difference/ratio (95%CI)**^a,c^
	Patients with lower-leg injury	Control subjects			
**Thrombin generation**	**(*N* = 89)**	**(*N* = 88)**			
ETP (nM IIa * min)	566.0 (202.2)	451.4 (239.4)	114.6 (48.8 to 180.3)	120.0 (55.0 to 185.1)	149.7 (67.7 to 231.6)
Thrombin peak (nM)	152.3 (59.4)	117.3 (67.4)	35.0 (16.1 to 53.8)	36.6 (17.8 to 55.3)	44.8 (21.1 to 68.5)
Lag time (min)	2.0 (0.5)	2.1 (0.6)	-0.1 (-0.3 to 0.0)	-0.1 (-0.3 to 0.0)	-0.1 (-0.3 to 0.1)
Velocity index (nM/min)	72.8 (33.8)	55.7 (37.5)	17.0 (6.4 to 27.6)	18.0 (7.4 to 28.6)	22.9 (9.4 to 36.4)
**In vivo markers**	**(*N* = 66)**	**(*N* = 28)**			
TAT complexes (ug/L)^a^	*5.7 (4.8 to 6.7)*^a^	*2.3 (2.1 to 2.5)*^a^	*2.5 (1.9 to 3.2)*^a^	*2.3 (1.7 to 3.0)*^a^	*2.3 (1.7 to 3.1)*^a^
Prothrombin fragment 1 + 2 (pmol/L)^a^	*260.7 (231.2 to 294.0)*^a^	*242.7 (218.6 to 269.3)*^a^	*1.1 (0.9 to 1.3)*^a^	*1.0 (0.8 to 1.2)*^a^	*1.0 (0.8 to 1.3)*^a^
Fibrinopeptide A (ng/mL)	143.9 (91.5)	95.1 (64.3)	48.8 (11.0 to 86.7)	43.8 (5.9 to 81.8)	57.2 (14.5 to 99.9)

^a^This symbol and italics indicate geometric means (with 95%CIs) and mean ratios in case of ln-retransformation due to skewed data

^b^Adjusted for age, sex, BMI and comorbidities

^c^Adjusted for age, sex, BMI, comorbidities and time of blood sampling (diurnal variation)

**Fig. 2:**
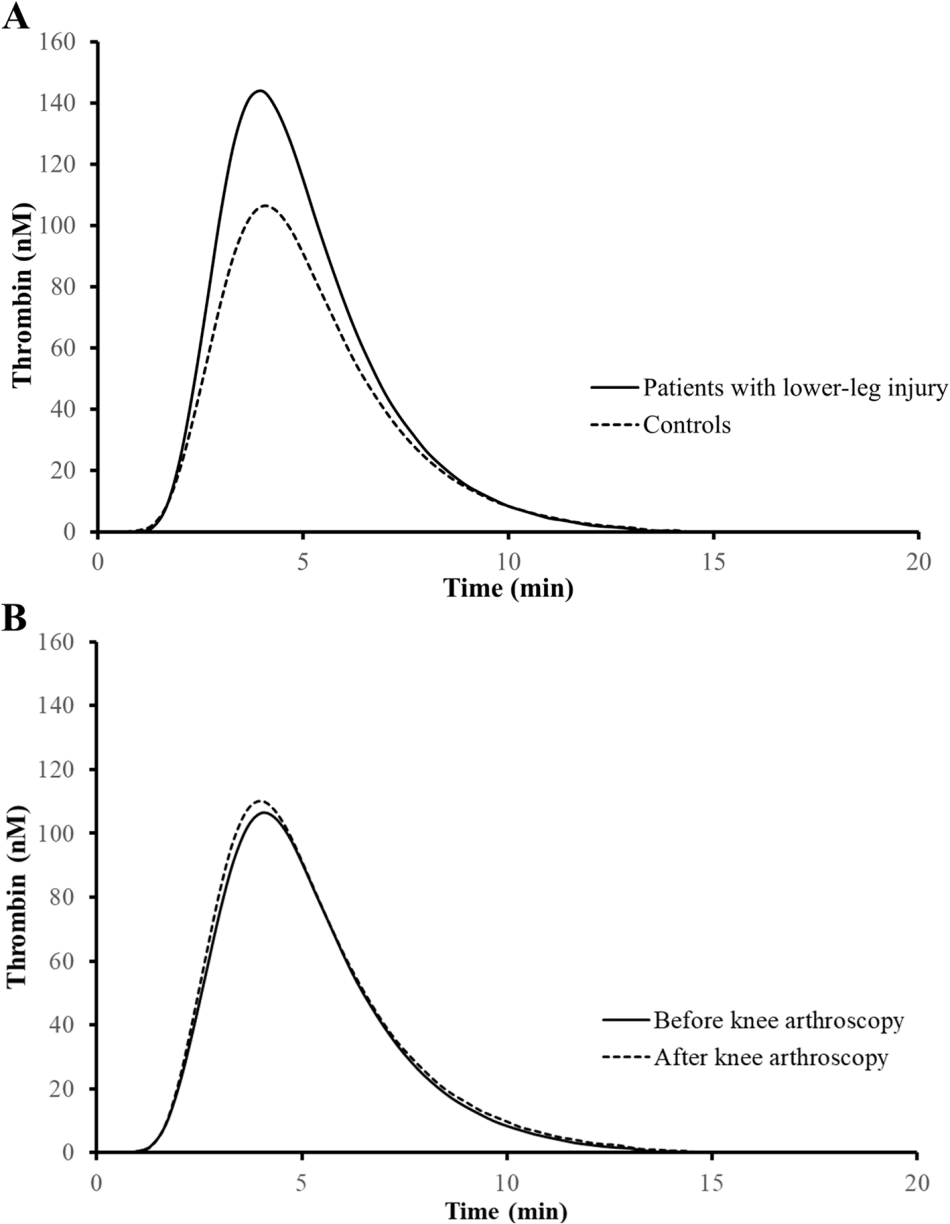
Mean values of thrombin generation over time. **A** Aim 1 (lower-leg injury patients versus controls); **B** Aim 2 (before versus after knee arthroscopy)

In comparison with control subjects, all thrombin generation parameters (except for lag time) were elevated in patients with lower-leg injury, also after adjusting for age, sex, BMI and comorbidity (Table 2). The mean differences were even larger after additional adjustment for time of blood sampling: ETP 149.7 nM IIa*min (95%CI 67.7 to 231.6), thrombin peak 44.8 nM IIa (95%CI 21.1 to 68.5), velocity Index 22.9 nM IIa/min (95%CI 9.4 to 36.4). In the thrombin generation curves in Fig. 2A these differences are visualized. Of the in vivo markers of coagulation activation, TAT and FPA were increased with a mean ratio of 2.3 (95%CI 1.7 to 3.0) and a mean difference of 57.2 ng/mL (95%CI 14.5 to 99.9), respectively. F1 + 2 levels, on the other hand were similar between the groups.

As shown in Table S1, FVIII had the largest impact on the effect of lower-leg injury on ETP, thrombin peak and velocity index, followed by FIX and fibrinogen and no effect of FXI.

### Aim 2: effect of knee arthroscopy on thrombin generation

#### Study population

Of the 85 knee arthroscopy patients, 52% was male and the median age was 51 years. 68% of the patients had a BMI ≥ 25 and 8% had at least one comorbidity. Most patients underwent a meniscectomy (63%) and general anesthesia was applied most of the time (66%). In almost all patients a thigh-tourniquet was applied during the procedure (98%) (Table 3).

**Table 3:** Aim 2: specifics of knee arthroscopic procedures

	**Knee arthroscopy patients, available for:**	
	Ex vivo thrombin generation (***N* = 85**)	In vivo thrombin generation (***N* = 28**)
**Type of procedure**		
Meniscectomy, *n (%)*	54 (63.5)	19 (67.9)
Diagnostic arthroscopy, *n (%)*	5 (5.9)	1 (3.6)
Removal of loose bodies, *n (%)*	3 (3.5)	0 (0.0)
Other, *n (%)*	9 (10.6)	4 (14.3)
Multiple procedures, *n (%)*	14 (16.5)	4 (14.3)
**ASA classification**^a^		
ASA I, *n (%)*	51 (62.2)	15 (55.6)
ASA II, *n (%)*	30 (36.6)	12 (44.4)
ASA III, *n (%)*	1 (1.2)	0 (0.0)
**Type of anesthesia**		
General,* n (%)*	56 (66.7)	16 (57.1)
Spinal, *n (%)*	28 (33.3)	12 (42.9)
**Use of thigh-tourniquet**		
Yes, *n (%)*	80 (97.6)	25 (96.2)
**Total duration of knee arthroscopy**^b^		
*Median in minutes (IQR)*	20.0 (16.0 – 27.0)	21.0 (16.0 – 27.3)
**Duration of surgery**^c^		
*Median in minutes (IQR)*	12.0 (9.0 – 16.0)	12.0 (10.0 – 14.8)

*IQR* Interquartile range (25^th^-75^th^ percentile)

^a^ASA: American Society of Anesthesiologists classification

^b^Total duration was from the time patient received anaesthesia to the time patient left the operating room

^c^Duration of surgery was defined from the time of incision to the time of wound closure

#### Outcomes

Mean levels of thrombin generation parameters in knee arthroscopy patients within four hours after surgery were: ETP 473.1 nM IIa*min, thrombin peak 121.0 nM IIa, lag time 2.0 min, velocity index 58.7 nM IIa/min (Table 4). Relative to preoperative mean levels, none of the parameters substantially changed. This was somewhat the same after adjusting for time of blood sampling, corresponding to the following paired mean changes: ETP 30.2 nM IIa*min (95%CI -20.5 to 80.9), thrombin peak 3.4 nM IIa (95%CI -10.7 to 17.4), lag time -0.1 min (95%CI -0.2 to 0.0), velocity index 0.9 nM IIa/min (95%CI -6.6 to 8.4). Accordingly, thrombin generation curves pre- and postoperatively were similar (Fig. 2B). Postoperative (geometric) mean levels of in vivo thrombin and fibrin generation markers were TAT 2.1 ug/L, F1 + 2 226.6 pmol/L and FPA 101.2 ng/mL. After adjusting for time of blood sampling, TAT levels were stable after arthroscopy, while F1 + 2 levels somewhat decreased and FPA slightly increased, as reflected by the following paired mean changes: TAT -0.2 ug/L (95%CI -0.6 to 0.2), F1 + 2 -12.2 pmol/L (95%CI -44.8 to 20.4), FPA 18.7 ng/mL (95%CI -14.1 to 51.4).

**Table 4:** Aim 2: ex vivo and in vivo thrombin generation in knee arthroscopy patients measured pre- and postoperatively

	**Mean (SD)**^b^		**Paired mean change (95%CI)**	**Paired mean change (95%CI)** ^c^
	Preoperative	Postoperative		
**Thrombin generation** (*N* = 85)^a^				
ETP (nM IIa * min)	444.4 (240.3)	473.1 (260.9)	28.7 (-4.1 to 61.5)	30.2 (-20.5 to 80.9)
Thrombin peak (nM)	116.0 (67.9)	121.0 (72.0)	5.0 (-4.0 to 13.9)	3.4 (-10.7 to 17.4)
Lag time (min)	2.2 (0.6)	2.0 (0.5)	-0.1 (-0.2 to 0.0)	-0.1 (-0.2 to 0.0)
Velocity index (nM/min)	55.5 (37.9)	58.7 (42.1)	3.2 (-2.0 to 8.3)	0.9 (-6.6 to 8.4)
**In vivo markers** (*N* = 28)				
TAT complexes (ug/L)^b^	*2.3 (2.1 to 2.5)*^b^	*2.1 (1.9 to 2.3)*^b^	-0.2 (-0.5 to 0.2)	-0.2 (-0.6 to 0.2)
Prothrombin fragment 1 + 2 (pmol/L)^b^	*242.7 (218.6 to 269.3)*^b^	*226.6 (201.4 to 255.1)*^b^	-14.9 (-38.4 to 8.5)	-12.2 (-44.8 to 20.4)
Fibrinopeptide A (ng/mL)	95.1 (64.3)	101.2 (64.0)	6.1 (-20.8 to 33.1)	18.7 (-14.1 to 51.4)

^a^Thrombin generation was not measured in the postoperative samples of three patients due to lack of plasma (*n* = 2) or presence of clots in plasma (*n* = 1)

^b^This symbol and italics indicate geometric means (with 95%CIs) in case of ln-retransformation due to skewed data

^c^Paired mean changes corrected for time of blood sampling (diurnal variation); calculated using LMM

## Discussion

In this study we found that lower-leg trauma was associated with enhanced ex vivo thrombin generation potential and enhanced in vivo thrombin and fibrin generation. In contrast, knee arthroscopy had no effect on either ex vivo or in vivo thrombin generation.

To our knowledge, the effect of lower-leg trauma on thrombin generation has not been described before. In contrast, the effect of major trauma on in vitro thrombin generation potential has been studied previously. These studies showed that severe injuries led to increased thrombin generation parameters (i.e. ETP, thrombin peak, velocity index) shortly after trauma [7, 21, 22]. To our knowledge it has not been demonstrated before that a minor trauma such as of the lower-leg, also leads to enhanced thrombin generation. The enhanced thrombin generating capacity following lower-leg trauma was partly explained by the elevated FVIII levels that we have found in our previous study [18]. FVIII is known as an acute phase reactant, i.e., its levels increase upon homeostatic disturbances such as inflammation [23–29]. It is known that (minor) trauma induces short-term hyperinflammation, during which remnants of tissue damage are removed by platelets and immune cells in order to prepare the tissue for repair [30]. It seems that damage associated molecular patterns (DAMP), which are released by damaged tissue and activated platelets, play a role in posttraumatic hyperinflammation as they activate innate inflammatory pathways [31]. Animal models showed that these pathways ultimately facilitate coagulation [32]. Another way in which thrombin generation is facilitated after lower-leg trauma is by procoagulant microvesicles, which are mostly released by activated platelets [33]. In another study, we demonstrated threefold increased plasma levels of procoagulant microvesicles after lower-leg trauma [34]. Procoagulant microvesicles express phosphotidylserine (PS) on their surface, which attracts positively-charged coagulation factors such as prothrombin, by which thrombin generation is facilitated [35, 36]. The resulting hypercoagulable state and the in vivo activation of coagulation associated with lower-leg trauma may explain the elevated VTE risk in these patients. It is unclear why F1 + 2 levels, in contrast to levels of TAT and FPA, are not increased in patients with lower-leg injury. Possibly, the longer half-time of F1 + 2 (approximately 90 min) as compared with that of TAT and FPA (around 15 and 3–5 min, respectively) plays a role in this [37–39].

It is unclear why thromboprophylaxis with LMWH does not reduce VTE risk in patients with lower-leg injury. It may be that the local exposure of tissue factor to the blood stream leads to strong activation of the coagulation system which overwhelms the anticoagulant activity of LMWH. It has been well established that anticoagulant therapy is not always able to fully block activation of coagulation, for example as recently evidenced by ongoing activation of coagulation in patients with COVID-19, despite prophylactic to even therapeutic doses of LMWH [40, 41]. Also in settings of therapeutic anticoagulation, for example during coronary surgery with the use of cardiopulmonary bypass, activation of coagulation may occur despite optimal anticoagulant therapy [42]. Future studies should assess effects of different types, intensities or durations of thromboprophylaxis on in vivo markers of thrombin generation in patients with lower-leg trauma, to assess whether such adaptations in anticoagulant therapy may reduce VTE risk.

Surprisingly, we did not observe any effect of knee arthroscopy on thrombin generation, neither ex vivo or in vivo. Although knee arthroscopy is a minimally invasive procedure that causes little iatrogenic tissue injury, we hypothesized to find some postoperative increase in thrombin generation as knee arthroscopy is associated with an increased thrombosis risk. To our knowledge, the association with thrombin generation has not been studied before, except in patients undergoing total knee arthroplasty [19, 43]. Knee replacement surgery is a far more invasive procedure than knee arthroscopy, causing extensive iatrogenic tissue injury. Furthermore, arthroscopy is a relatively short procedure (median 20 min in our study). These factors could explain the lack of detectable changes in thrombin generation parameters following arthroscopy. Hence, another mechanism may ultimately lead to the increased VTE risk in these patients. The use of thigh-tourniquet, which is applied above the knee in order to block the arterial blood supply of the leg and create a “dry surgical field”, might have a role in this mechanism. A tourniquet causes hypoxia and stasis in the leg which could result in delayed activation of coagulation via HIF-1 and Egr-1 pathways, resulting in endothelial activation and release of procoagulant microvesicles [44]. This hypothesis should be tested in future studies.

An important strength of our study was that we measured thrombin generation in blood samples which were collected shortly after exposure to lower-leg trauma (i.e., in the acute phase after trauma) and knee arthroscopy (i.e., within a few hours after surgery), but before administration of thromboprophylaxis. This enabled us to study acute activation of coagulation without interference of exogenous anticoagulants in an unselected population. There were also limitations. First, not all patients with lower-leg injury presented at the Emergency Department immediately after trauma, i.e., around 70% of the patients presented in the first 24 h. However, ETP did not correlate with time between trauma and blood sampling, i.e., ETP remained stable in time. Therefore, we concluded that it was unlikely that a delay in sampling affected our results. Second, it is possible that we measured thrombin generation too early after knee arthroscopy to observe a strong effect, and that an extra measurement at (for instance) 24 h postoperatively would have been more informative.

Our study results indicate that lower-leg trauma initiates a hypercoagulable state in contrast to knee arthroscopy. Since there is elevated VTE risk in both situations, these findings suggest that there are distinct pathways towards the development of VTE. However, further research is necessary, where it should be established whether enhanced thrombin generation potential and elevated levels of in vivo thrombin generation markers are elevated on the long-term (for which longitudinal data are necessary), and if and for how long these elevations are associated with increased VTE risk.. For knee arthroscopy, it should be explored whether there is a delayed activation of coagulation after the surgery, possibly associated with tourniquet use. Thereafter, the influence of prophylactic exogenous anticoagulants on coagulation should be studied in both populations. In this way, our hypothesis that prophylaxis with anticoagulants, as currently applied, insufficiently downregulates clot formation can be tested. An approach to improve thromboprophylaxis strategy in both patient groups is to identify potential new treatment options that target specific thrombogenic mechanisms. Our data suggest that thrombin generation tendency is enhanced in patients with lower-leg trauma, which may necessitate different or more intensified anticoagulant regimens.

In conclusion, lower-leg injury strongly affects both ex vivo thrombin generation (potential) and in vivo thrombin generation. Hence, enhanced thrombin generation triggered by the lower-leg injury most likely plays a key role in the mechanism of thrombus formation in patients with lower-leg cast immobilization. Knee arthroscopy, on the other hand, did not appear to have an effect on thrombin generation. This may imply that the pathogenesis of VTE is different in both situations.

## Supplementary Information

**Additional file 1.**

## Data Availability

Data will be available (conditional on agreement on privacy matters and appropriate use of the data) upon request at the data-manager of the Department of Clinical Epidemiology of Leiden University Medical Center: Ingeborg de Jonge, e-mail: i.de_jonge@lumc.nl.

## Abbreviations

95%CI: 95% Confidence interval
BMI: Body mass index
CAT: Calibrated Automated Thrombography
COPD: Chronic obstructive pulmonary disease
ETP: Endogenous thrombin potential
F1 + 2: Prothrombin fragment 1 + 2
FVIII/IX/XI: Factor VIII/IX/XI
FPA: Fibrinopeptide A
LMM: Linear mixed models
LMWH: Low-molecular-weight-heparin
Ln: Natural logarithm
SD: Standard deviation
TAT: Thrombin-antithrombin
TGA: Thrombin generation assay
VTE: Venous thromboembolism

## References

[1] Alotaibi GS, Wu C, Senthilselvan A, McMurtry MS (2016). Secular trends in incidence and mortality of acute venous thromboembolism: the AB-VTE population-based study. Am J Med.

[2] Piazza G, Goldhaber SZ (2011). Chronic thromboembolic pulmonary hypertension. N Engl J Med.

[3] Baldwin MJ, Moore HM, Rudarakanchana N, Gohel M, Davies AH (2013). Post-thrombotic syndrome: a clinical review. J Thromb Haemost.

[4] Nemeth B, Cannegieter SC (2019). Venous thrombosis following lower-leg cast immobilization and knee arthroscopy: from a population-based approach to individualized therapy. Thromb Res.

[5] van Adrichem RA, Nemeth B, Algra A, le Cessie S, Rosendaal FR, Schipper IB (2017). Thromboprophylaxis after knee arthroscopy and lower-leg casting. N Engl J Med.

[6] Kahn SR, Shivakumar S (2020). What's new in VTE risk and prevention in orthopedic surgery. Res Pract Thromb Haemost.

[7] Park MS, Spears GM, Bailey KR, Xue A, Ferrara MJ, Headlee A (2017). Thrombin generation profiles as predictors of symptomatic venous thromboembolism after trauma: a prospective cohort study. J Trauma Acute Care Surg.

[8] Park MS, Xue A, Spears GM, Halling TM, Ferrara MJ, Kuntz MM (2015). Thrombin generation and procoagulant microparticle profiles after acute trauma: a prospective cohort study. J Trauma Acute Care Surg.

[9] Davie EW, Kulman JD (2006). An overview of the structure and function of thrombin. Semin Thromb Hemost.

[10] Mann KG, Butenas S, Brummel K (2003). The dynamics of thrombin formation. Arterioscler Thromb Vasc Biol.

[11] Capecchi M, Scalambrino E, Griffini S, Grovetti E, Clerici M, Merati G (2021). Int J Lab Hematol.

[12] Ota S, Wada H, Abe Y, Yamada E, Sakaguchi A, Nishioka J (2008). Elevated levels of prothrombin fragment 1 + 2 indicate high risk of thrombosis. Clin Appl Thromb Hemost.

[13] Prisco D (1990). Markers of increased thrombin generation. Res Clin Lab.

[14] Hemker HC, Giesen P, Al Dieri R, Regnault V, de Smedt E, Wagenvoord R (2003). Calibrated automated thrombin generation measurement in clotting plasma. Pathophysiol Haemost Thromb.

[15] Lisman T, Adelmeijer J (2020). Preanalytical variables affect thrombomodulin-modified thrombin generation in healthy volunteers. Thromb Res.

[16] Gennarelli T, Wodzin E (2008). The Abbreviated Injury Scale 2005. Update 2008. American Association for Automotive Medicine (AAAM).

[17] Orsi FA, Biedermann JS, Kruip M, van der Meer FJ, Rosendaal FR, van Hylckama Vlieg A, et al. Rosuvastatin use reduces thrombin generation potential in patients with venous thromboembolism: a randomized controlled trial. J Thromb Haemost. 2019;17(2):319–28. 10.1111/jth.14364 PMC6850636 30565854

[18] Touw CE, Nemeth B, Rondon AMR, van Adrichem RA, Lisman T, Versteeg HH (2022). Lower-leg injury and knee arthroscopy have distinct effects on coagulation. Blood Adv.

[19] Gionis MN, Ioannou CV, Katsamouris AN, Katonis P, Balalis K, Sfyridaki K (2013). The study of the thrombin generation mechanism and the effect of low molecular weight heparin as thromboprophylaxis in patients undergoing total knee and hip replacement. Thromb Res.

[20] Lee SY, Niikura T, Iwakura T, Sakai Y, Kuroda R, Kurosaka M (2017). Thrombin-antithrombin III complex tests. J Orthop Surg (Hong Kong).

[21] Selby R, Geerts W, Ofosu FA, Craven S, Dewar L, Phillips A (2009). Hypercoagulability after trauma: hemostatic changes and relationship to venous thromboembolism. Thromb Res.

[22] Voils SA, Lemon SJ, Jordan J, Riley P, Frye R (2016). Early thrombin formation capacity in trauma patients and association with venous thromboembolism. Thromb Res.

[23] Pabinger I, Ay C (2009). Biomarkers and venous thromboembolism. Arterioscler Thromb Vasc Biol.

[24] Kawecki C, Lenting PJ, Denis CV (2017). von Willebrand factor and inflammation. J Thromb Haemost.

[25] Cucuianu M, Pleşca L, Bodizs G, Colhon D, Brudaşcă I (1996). Acute phase reaction and the hemostatic balance. Rom J Intern Med.

[26] Jain S, Gautam V, Naseem S (2011). Acute-phase proteins: as diagnostic tool. J Pharm Bioallied Sci.

[27] Begbie M, Notley C, Tinlin S, Sawyer L, Lillicrap D (2000). The Factor VIII acute phase response requires the participation of NFkappaB and C/EBP. Thromb Haemost.

[28] Kerr R, Stirling D, Ludlam CA (2001). Interleukin 6 and haemostasis. Br J Haematol.

[29] Greven J, Pfeifer R, Zhi Q, Pape HC (2018). Update on the role of endothelial cells in trauma. Eur J Trauma Emerg Surg.

[30] Jorch SK, Kubes P (2017). An emerging role for neutrophil extracellular traps in noninfectious disease. Nat Med.

[31] Zhang Q, Raoof M, Chen Y, Sumi Y, Sursal T, Junger W (2010). Circulating mitochondrial DAMPs cause inflammatory responses to injury. Nature.

[32] Vogel S, Bodenstein R, Chen Q, Feil S, Feil R, Rheinlaender J (2015). Platelet-derived HMGB1 is a critical mediator of thrombosis. J Clin Investig.

[33] Żmigrodzka M, Guzera M, Miśkiewicz A, Jagielski D, Winnicka A (2016). The biology of extracellular vesicles with focus on platelet microparticles and their role in cancer development and progression. Tumour Biol.

[34] Touw CE, Nemeth B, Lijfering WM, van Adrichem RA, Wilsgård L, Latysheva N (2022). Effect of lower-leg trauma and knee arthroscopy on procoagulant phospholipid-dependent activity. Res Pract Thromb Haemost.

[35] Freyssinet JM, Toti F (2010). Formation of procoagulant microparticles and properties. Thromb Res.

[36] Grover SP, Mackman N (2018). Tissue factor: an essential mediator of hemostasis and trigger of thrombosis. Arterioscler Thromb Vasc Biol.

[37] Páramo JA, Orbe J, Beloqui O, Benito A, Colina I, Martinez-Vila E (2004). Prothrombin fragment 1+2 is associated with carotid intima-media thickness in subjects free of clinical cardiovascular disease. Stroke.

[38] Lippi G, Cervellin G, Franchini M, Favaloro EJ (2010). Biochemical markers for the diagnosis of venous thromboembolism: the past, present and future. J Thromb Thrombolysis.

[39] Nossel HL, Yudelman I, Canfield RE, Butler VP, Spanondis K, Wilner GD (1974). Measurement of fibrinopeptide A in human blood. J Clin Investig.

[40] Blasi A, von Meijenfeldt FA, Adelmeijer J, Calvo A, Ibañez C, Perdomo J (2020). In vitro hypercoagulability and ongoing in vivo activation of coagulation and fibrinolysis in COVID-19 patients on anticoagulation. J Thromb Haemost.

[41] von Meijenfeldt FA, Havervall S, Adelmeijer J, Lundström A, Rudberg AS, Magnusson M (2021). Prothrombotic changes in patients with COVID-19 are associated with disease severity and mortality. Res Pract Thromb Haemost.

[42] Brister SJ, Ofosu FA, Buchanan MR (1993). Thrombin generation during cardiac surgery: is heparin the ideal anticoagulant?. Thromb Haemost.

[43] Su EP, Mount LE, Nocon AA, Sculco TP, Go G, Sharrock NE (2018). Changes in markers of thrombin generation and interleukin-6 during unicondylar knee and total knee arthroplasty. J Arthroplasty.

[44] Bovill EG, van der Vliet A (2011). Venous valvular stasis-associated hypoxia and thrombosis: what is the link?. Annu Rev Physiol.

