# Supplementary material for: Enhanced in vivo and ex vivo thrombin generation after lower-leg trauma, but not after knee arthroscopy
Source: Thromb J. 2023 Apr 28;21:49. doi: 10.1186/s12959-023-00493-4 (PMC10139833; doi:10.1186/s12959-023-00493-4)
Supplement: Supplementary file 1 — Additional file 1. [file 12959_2023_493_MOESM1_ESM.docx]

**Enhanced in vivo and ex vivo thrombin generation after lower-leg trauma, but not after knee arthroscopy – SUPPLEMENT**

**Statistical analyses – Data transformation using natural logarithms**

In case of not normally distributed data, transformations were performed using natural logarithms (*ln*). In order to obtain geometric means, the *ln* of all values of the variable concerned (in this example referred to as ‘*X*’) were computed, after which the mean of *ln-X* was calculated and retransformed by calculating *e^(mean ln-X)*, resulting in the geometric mean. In linear regression models, *ln-X* was included as the dependent variable. Since the outcome estimate of linear regression is mean difference, for ln-transformed variables, this involves *ln(b) – ln(a)*, which equals *ln(b/a)*. Therefore, by retransforming the outcome of linear regression, i.e. *e^(ln(b/a))*, a mean ratio (i.e. *b/a*) is obtained. The geometric means and mean ratios were obtained with their 95% confidence intervals (95%CIs).

**Fig S1** – Aim 1: Distribution of ETP stratified for lower-leg injury type depicted with medians (lines).


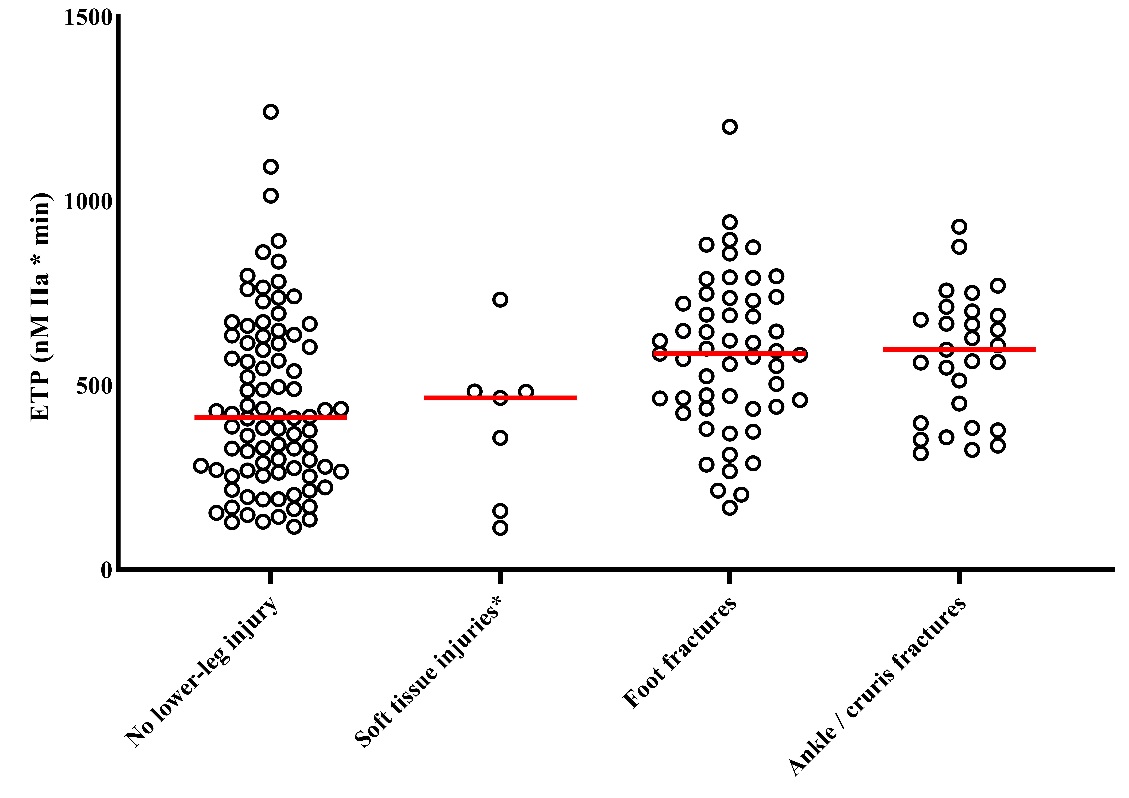


*Soft tissue injury includes Achilles’ tendon rupture, ankle distortion and contusion.

**Fig S2** – Aim 1: ETP (A) and TAT (B) plotted against time between lower-leg trauma and blood draw.

**A**
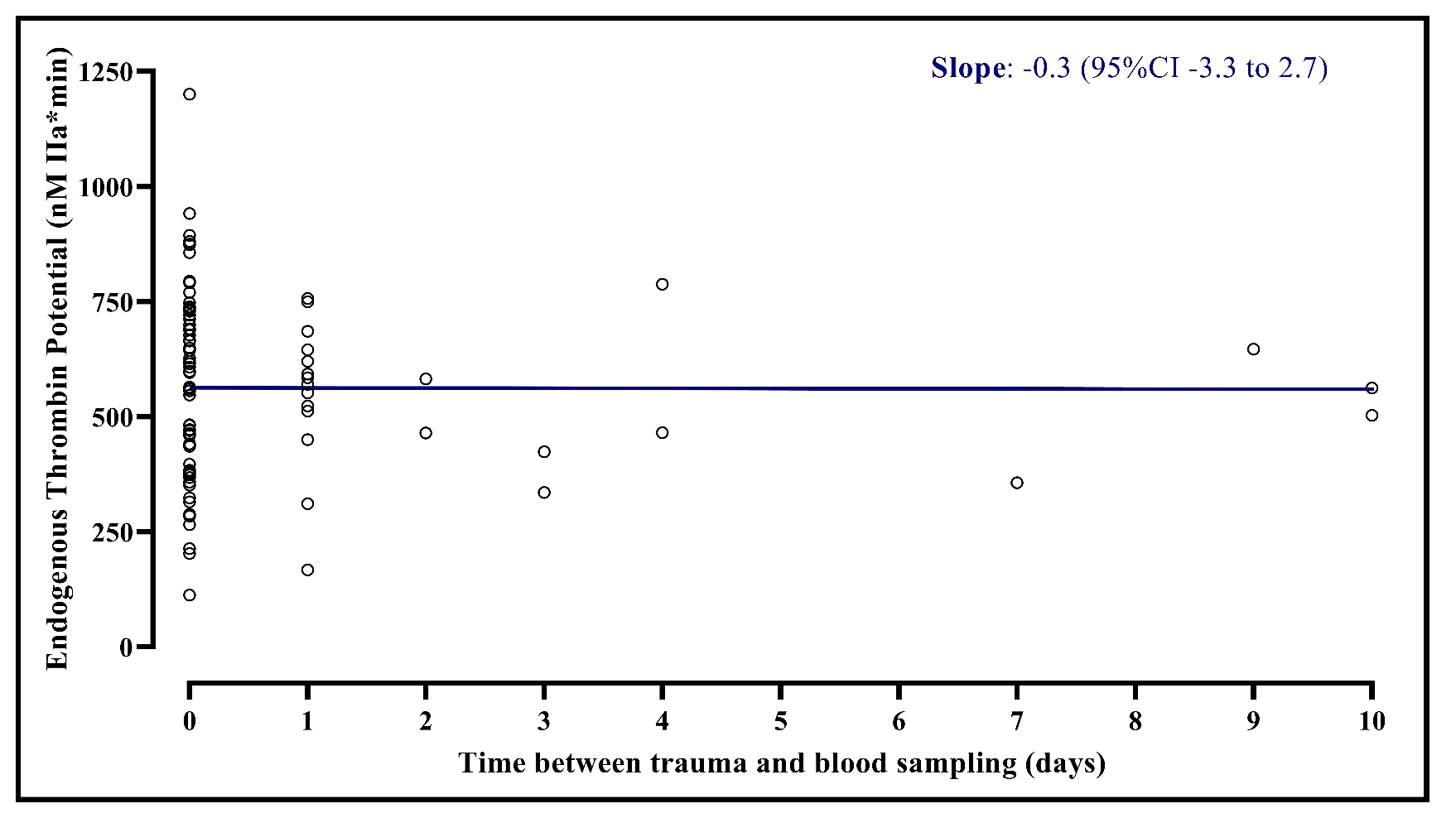


**B**
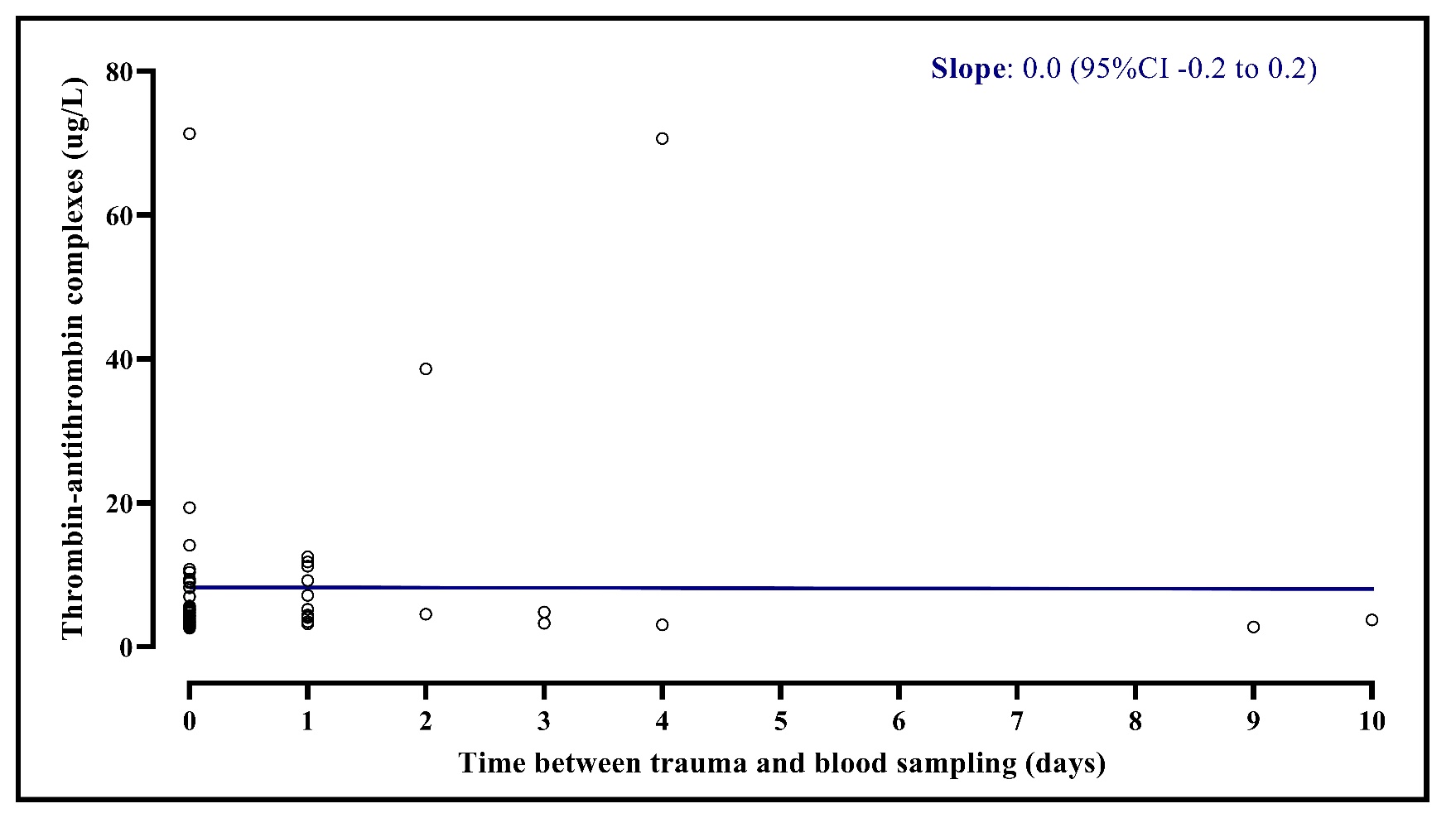


**Table S1** – Aim 1: Effect of lower-leg injury on ETP adjusted for coagulation factor levels.

|  | Mean difference (95%CI) | |
| --- | --- | --- |
|  | Crude | Adjusted ^a^ |
| ETP (nM IIa * min) |  |  |
| No coagulation factor | 114.6 (48.8 to 180.3) | 150.5 (70.2 to 230.8) |
| + Fibrinogen | 105.2 (39.8 to 170.5) | 137.5 (57.6 to 217.5) |
| + FVIII | 87.5 (16.6 to 158.5) | 120.3 (35.2 to 205.4) |
| + FIX | 104.4 (37.0 to 171.9) | 135.8 (54.5 to 217.1) |
| + FXI | 113.3 (47.3 to 179.3) | 152.9 (71.7 to 234.1) |
| + Fibrinogen, FVIII, FIX, FXI | 78.0 (6.1 to 150.0) | 100.2 (16.1 to 184.3) |
| Thrombin peak (nM) |  |  |
| No coagulation factor | 35.0 (16.1 to 53.8) | 44.6 (21.4 to 67.8) |
| + Fibrinogen | 32.5 (13.7 to 51.3) | 41.2 (18.0 to 64.3) |
| + FVIII | 23.9 (3.7 to 44.0) | 32.3 (8.0 to 56.6) |
| + FIX | 31.3 (12.0 to 50.6) | 39.5 (16.1 to 62.9) |
| + FXI | 34.2 (15.3 to 53.1) | 44.4 (20.9 to 67.9) |
| + Fibrinogen, FVIII, FIX, FXI | 21.6 (1.1 to 42.1) | 27.0 (2.9 to 51.1) |
| Velocity index (nM/min) |  |  |
| No coagulation factor | 17.0 (6.4 to 27.6) | 22.5 (9.3 to 35.7) |
| + Fibrinogen | 15.8 (5.2 to 26.4) | 20.8 (7.6 to 34.0) |
| + FVIII | 10.2 (-1.1 to 21.4) | 14.7 (1.0 to 28.4) |
| + FIX | 14.7 (3.8 to 25.5) | 19.3 (6.0 to 32.5) |
| + FXI | 16.6 (6.0 to 27.2) | 22.3 (8.9 to 35.7) |
| + Fibrinogen, FVIII, FIX, FXI | 8.7 (-2.8 to 20.2) | 11.8 (-1.8 to 25.4) |

^a^ Adjusted for age, sex and time of blood sampling (diurnal variation).
